# Impact of metformin treatment on cobalamin status in persons with type 2 diabetes

**DOI:** 10.1093/nutrit/nuad045

**Published:** 2023-05-11

**Authors:** Sundus Fituri, Zoha Akbar, Vijay Ganji

**Affiliations:** Human Nutrition Department, College of Health Sciences, QU Health, Qatar University, Doha, Qatar; Human Nutrition Department, College of Health Sciences, QU Health, Qatar University, Doha, Qatar; Human Nutrition Department, College of Health Sciences, QU Health, Qatar University, Doha, Qatar

**Keywords:** cobalamin, intrinsic factor, metformin, type 2 diabetes, vitamin B_12_

## Abstract

Over the last decades, low vitamin B_12_ status has been reported in individuals with type 2 diabetes mellitus (T2DM). Metformin, the first-line therapy for lowering blood glucose, is the main driving factor behind this association. Although the relationship between vitamin B_12_ deficiency and metformin is well established, results of studies on the exact effect of the dose and duration of the therapy remain inconsistent. Additionally, a lack of consensus on the definition of vitamin B_12_ deficiency adds to the conflicting literature. The objectives of this review were to analyze and synthesize the findings on the effects of metformin dose and duration on vitamin B_12_ status in patients with T2DM and to outline the potential mechanisms underlying metformin’s effect on vitamin B_12_. Metformin therapy has adversely affected serum vitamin B_12_ concentrations, a marker of vitamin B_12_ status. The metformin usage index (a composite score of metformin dose and duration) might serve as a potential risk assessment tool for vitamin B_12_ screening in patients with T2DM. Considering the health implications of suboptimal vitamin B_12_ status, vitamin B_12_ concentrations should be monitored periodically in high-risk patients, such as vegans who are receiving metformin therapy for T2DM. Additionally, it is prudent to implement lifestyle strategies concurrent with metformin therapy in individuals with T2DM, promoting an overall synergistic effect on their glycemic control.

## INTRODUCTION

Vitamin B_12_ (cobalamin), a hydrophilic vitamer, is derived primarily from foods of animal origin such as dairy, meat, and eggs.^1^ Several foods such as breakfast cerelas, bread, and snack bars are fortified with vitamin B_12_, providing an alternate source of vitamin B_12_ for vegans and vegetarians. In food, vitamin B_12_ is bound to protein and requires cleaving into its free form by hydrochloric acid in the gastric lumen before absorption.^2^ After cleavage, haptocorrin, a glycoprotein, binds to vitamin B_12_. This bound form is then cleaved by pancreatic proteases in the duodenum. The resulting free cobalamin binds to a carrier protein called intrinsic factor (IF). IF facilitates cobalamin absorption in the small intestine and its subsequent release into circulation via the intestinal brush border. Vitamin B_12_ is delivered to the liver and peripheral tissues attached to the transport proteins haptocorrin or transcobalamin II. Haptocorrin-bound vitamin B_12_ is delivered primarily to the liver because of a lack of haptocorrin receptors on peripheral cells, and accounts for 70%–80% of the circulating vitamin B_12_. Transcobalamin II chiefly supplies vitamin B_12 _to all cells, though only 20%–30% of serum cobalamin is bound to transcobalamin II as holotranscobalamin in the blood.^3^

In adults with healthy gastrointestinal function, vitamin B_12_ bioavailability ranges from 4.5% to 83%, depending on the food source and saturation of digestive proteins. Bioavailability may be lower in those with less-than-optimal gastric acid, IF, and transport carrier protein.^1^^,^^3^^,^^4^ Vitamin B_12_ acts as a coenzyme for 2 mammalian enzymes. Methionine synthase remethylates homocysteine to methionine, generating tetrahydrofolate for the synthesis of nucleic acids. It also holds neurological significance through the maintenance of the myelin sheath in nerve cells via methionine and subsequent *S*-adenosylmethionine production.^5^ In the mitochondria, methylmalonyl coenzyme A (CoA) is converted to succinyl CoA, which is catalyzed by methylmalonyl CoA mutase. This enzyme is dependent on *S*-adenosylcobalamin, a vitamin B_12_ coenzyme. This biochemical reaction is part of the catabolic pathways of methionine, isoleucine, valine, threonine, cholesterol side chains, and odd-numbered carbon chain fatty acids. Suboptimal cobalamin concentrations lead to macrocytosis, neurological changes, hyperhomocysteinemia (a risk factor for cardiovascular disease), and elevated concentrations of methylmalonic acid (MMA), a byproduct of an excessive buildup of methylmalonyl CoA.^6–8^

The deficiency of cobalamin is diagnosed using serum cobalamin concentrations with varying cutoff values based on the testing laboratory’s diagnostic criteria. No gold standard exists for measuring cobalamin status, but serum cobalamin concentrations <148 pmol/L are used for diagnosing vitamin B_12_ deficiency.^9^ Although sensitive, this test has a low specificity.^10^ Serum vitamin B_12_ tests measure total serum cobalamin bound to haptocorrin and transcobalamin II and do not reflect true tissue stores of cobalamin. Tests measuring holotranscobalamin (cobalamin-bound transcobalamin II), the bioactive vitamin B_12_ form, can be used as an early indicator of vitamin B_12_ malabsorption because of its short half-life.^11^ Assessing vitamin B_12_ status using 1 functional marker of vitamin B_12_ deficiency (elevated total homocysteine [tHcy] or MMA) and 1 marker of serum cobalamin (total serum cobalamin or holotranscobalamin) has been suggested to accurately identify cobalamin deficiency in symptomatic individuals with normal serum cobalamin status.^12^^,^^13^

Limited dietary intake and absorption, among other causes, may affect vitamin B_12_ status and lead to its deficiency (Table 1).^9^^,^^14^^,^^15^ Symptoms of deficiency can manifest as fatigue, neuropathy, anemia, pale skin, glossitis, hyperpigmentation of the skin, and neurological impairment.^16–18^ The oral hypoglycemic agent metformin has been implicated in vitamin B_12_ deficiency.

**Table 1:** Etiology and pathogenesis of vitamin B12 deficiency

Etiology	Pathogenesis of cobalamin deficiency
Autoimmune diseases	Low IF synthesis due to IF antibodies (in pernicious anemia and Sjögren’s syndrome) Type 1 diabetes mellitus, hypothyroidism
Dietary	Low intake of B_12_-rich foods (eg, women of childbearing age who are of South Asian origin; excessive alcohol intake; vegan and vegetarian diets)
Gut disorder–associated malabsorption	Impaired B_12_ absorption due to (1) low IF synthesis in Crohn’s disease, celiac disease, and gastritis due to gut mucosal atrophy; (2) uptake of B_12_ by bacteria in the small intestine due to bacterial overgrowth; (3) reduced pancreatic enzyme and subsequent impaired proteolysis in pancreatic disorders (chronic pancreatitis)
Medication-associated malabsorption	Extended use of gastric pH–lowering medications such as antacids, H2 receptor antagonists, and proton pump inhibitors; use of oral contraceptives; metformin
Posturgical malabsorption	Decreased absorption due to ileal resection Total or partial gastrectomy Bariatric surgery
Genetic or other causes	Impaired B_12_ cellular transport (in transcobalamin II deficiency) Family history, older age, chronic alcoholism, HIV

*Abbreviations:* B_12_, vitamin B_12_; H2, histamine receptor-2; HIV, human immunodeficiency virus; IF, intrinsic factor.

Metformin is widely used as the first choice medication for the treatment of type 2 diabetes mellitus (T2DM). It is considered safe, economical, and efficacious in improving glycated hemoglobin concentrations.^19^ T2DM is a global health concern with a prevalence of 462 million cases worldwide.^20^ In this narrative review, the evidence on cobalamin status in metformin-treated patients with T2DM was analyzed and synthesized.

## LITERATURE SELECTION

Relevant studies on the association between metformin and vitamin B_12_ status were searched in the PubMed, Cochrane, SCOPUS, and Embase databases. Studies published in English after July 2013 were included because previous research had already been systematically reviewed and meta-analyzed.^21–23^ The search was not limited to study design, though studies with larger sample sizes were given more priority. Studies assessing the specific influence of the dose and duration of metformin were also prioritized. Studies with sample sizes smaller than 250 were excluded. The following search items were used: (((Metformin/) OR (Diabetes Mellitus/)) OR (Diabetes Mellitus, Type 2/)) AND (((Vitamin B 12/) OR (Vitamin B 12 Deficiency/)) OR (Cobalamin)). Some articles were identified through manual searching and reference tracking.

### Metformin-associated vitamin B_12_ deficiency: analysis of clinical evidence

A summary of selected studies on the relationship between metformin treatment and cobalamin status is presented in Table 2.^24–33^ The relationship between metformin therapy and cobalamin deficiency has long been documented.^34^ First published in the late 1960s, reports of this relationship demonstrated a decreased absorption of cobalamin in patients who were taking metformin for T2DM.^35^^,^^36^ In a large systematic review of research conducted up until 2013, the majority of included observational studies (59%) revealed significantly lower concentrations of vitamin B_12_ in patients with T2DM undergoing metformin therapy compared with those who were not taking metformin.^21^ In that study, a meta-analysis of 4 intervention trials showed a mean reduction of 57 pmol/L in vitamin B_12_ concentrations after 6 weeks to 3 months of metformin use (weighted mean difference, –57.1; 95%CI, –35.5, –78.8).^21^ Because of significant heterogeneity between the studies (*I*^2^ = 72%), the authors suggested interpreting the results with caution.

**Table 2:** Summary of studies on vitamin B12 status in relation to metformin therapy in people with type 2 diabetes mellitus

Reference	Study design, country, and sample size	Sample characteristics	Vitamin B_12_ (primary) or MMA (secondary) assessments^a^	Main findings and conclusions
de Groot-Kamphuis et al (2013)^24^	Cross-sectional Netherlands *N* = 298	Patients with T2DM receiving metformin (no specific duration) Mean age: 64.8 y	Deficiency: serum B_12_ <150 pmol/L	Each 100 mg of metformin dose increased odds for B_12_ deficiency by 8% (*P* = 0.014). Duration of metformin use had no effect (*P* = 0.78).
Beulens et al (2015)^25^	Cross-sectional Netherlands *N* = 550	Patients with T2DM receiving metformin (average dose, 1306 mg/d; average duration, 64 mo) Mean age: 61.6 y	Deficiency: serum B_12_ <148 pmol/L	Each increase of 1 mg/d in metformin dose decreased serum B_12_ and holotranscobalamin by 0.042 pmol/L (*P* < 0.001) and 0.012 pmol/L (*P* < 0.001), respectively.
Yousef Khan et al (2021)^26^	Cross-sectional Qatar *N* = 3124	Patients with T2DM receiving metformin for ≥3 mo Mean age: 56.6 y	Deficiency: serum B_12_ ≤145 pmol/L	A negative relation between serum B_12_ level and metformin dose (*r* = –0.32; *P* = 0.01). No relation between cobalamin and metformin use duration (*r* = 0.02; *P* = 0.1).
Miyan et al (2020)^27^	Cross-sectional Pakistan *N* = 932	Patients with T2DM receiving metformin for >2 y (69.2%) compared with Patients with T2DM not receiving metformin (30.8%)	Normal: serum B_12_ >221.4 pmol/L Insufficient: serum B_12_ 147.6–221.4 pmol/L Deficient: serum B_12_ <147.6 pmol/L	B_12_ deficiency was higher in users of metformin (3.9%) than in non-users of metformin (2.1%).
Kim et al (2019)^28^	Cross-sectional South Korea *N* = 1111	Patients with T2DM receiving metformin ≥6 mo Mean age: 59.5 y	Deficiency: serum B_12_ <221.4 pmol/L	For every 1-mg increase in daily metformin dose, serum B_12_ level decreased by 0.1 pmol/L (*P* < 0.001).
Ko et al (2014)^29^	Cross-sectional South Korea *N* = 799	Patients with T2DM receiving metformin for ≥3 mo Mean age: 59 y	Deficiency: serum B_12_ ≤221.4 pmol/L (without folate deficiency)	OR for B_12_ deficiency at higher doses of metformin (≥2000 mg vs ≤1000 mg) was 3.8 (*P* < 0.001). Compared with metformin use <4 y, OR for B_12_ deficiency for metformin use ≥10 y increased to 9.21 (*P* < 0.001)
Martin et al (2021)^30^	Longitudinal (retrospective cohort) United States *N* = 13 489	Patients with T2DM receiving metformin for >1 y Age range: 50–64 y	Deficiency: serum B_12_ <132.84 pmol/L	3.3% of metformin users tested (44.9%) were B_12_ deficient (vs 2.2% of comparisons). Average time between start of metformin therapy and incidence of B_12_ deficiency was 5.3 y.
Shivaprasad et al (2020)^31^	Longitudinal (prospective cohort) India *N* = 2887	Patients with T2DM receiving metformin and patients with T2DM not using metformin Age range: 20–65 y	Absolute deficiency: serum B_12_ <147.6 pmol/L Borderline deficiency: serum B_12_ 147.6–221.4 pmol/L Normal: serum B_12_ >221.4 pmol/L	Absolute B_12_ deficiency was higher in metformin users (24.5%) than in nonusers of metformin (17.3%). Odds for B_12_ deficiency were 6.74 times higher (*P* < 0.001) in metformin users with MUI >15 compared with non-metformin users.
Out et al (2018)^32^	Post hoc analysis of RCT (HOME study) Netherlands *N* = 390	Patients with T2DM receiving 850 mg metformin or placebo 1–3 times/d (in addition to insulin therapy) for 4.3 y Age range: 30–80 y	Serum MMA, a marker of B_12_ deficiency	Compared with placebo, metformin therapy increased serum MMA level by 0.039 μmol/L (*P* = 0.001).
Aroda et al (2016)^33^	Post hoc analysis of RCT, multicenter study (DPP study) United States *n* = 1073 taking metformin *n* = 1082 receiving placebo	Individuals with high risk for T2DM (increased fasting glucose, decreased glucose tolerance, and increased body adiposity) taking 850 mg metformin twice daily for 3.2 y (extended for an additional 9 y) or placebo for 3.2 y Age: ≥25 y	Low serum B_12_, <150 pmol/L Borderline-low serum B_12_, 150–220 pmol/L	B_12_ deficiency was higher in metformin users (4.3%) than in the placebo group (2.3%) at 5 y (*P* = 0.02). For every year of metformin use, the odds for cobalamin deficiency increased by 13%.

a Conversion: 1 pmol/L = 1.355 pg/mL.

*Abbreviations:* B_12_, vitamin B_12_; DPP, Diabetes Prevention Program; HOME, Hyperinsulinemia: The Outcome of its Metabolic Effects, a randomized controlled trial; MMA, methylmalonic acid; MUI, metformin usage index; OR, odds ratio; RCT, randomized controlled trial; T2DM, type 2 diabetes mellitus.

Since 2013, several observational and interventional studies continued to support the relationship between metformin therapy and serum cobalamin concentrations.^24–33^ However, the focus of the literature began to shift toward determining the impact of metformin dosage and duration of treatment of metformin on serum cobalamin concentrations. One large cross-sectional study in Korea demonstrated that for every 1-mg increase in daily metformin dose, vitamin B_12_ concentrations decreased by 0.142 pg/mL (95%CI, –0.169, –0.114).^28^ When compared with a group taking <1000 mg metformin daily, groups taking 1500–2000 mg or ≥2000 mg had odds ratios (ORs) of 3.34 (95%CI, 1.95, 5.75), and 8.67 (95%CI, 4.68, 16.06), respectively.^28^ In another study, the OR for vitamin B_12_ deficiency at higher doses of metformin (≥2000 mg vs ≤1000 mg) increased to 3.8 (95%CI, 1.82, 7.92).^29^ On the contrary, after adjustment for confounding factors, some studies found no effect of metformin dose on serum concentrations of vitamin B_12_.^33^^,^^37^^,^^38^

Similarly, findings on the effect of metformin duration on vitamin B_12_ status remain inconsistent. One large, retrospective cohort of adult patients (n = 13 489) found that the average time to develop vitamin B_12_ deficiency after metformin initiation was 5.3 years.^30^ A post hoc analysis of a randomized control trial found that for every year of metformin use, the OR of cobalamin deficiency increased by 13% (OR, 1.13; 95%CI, 1.06, 1.20).^33^ In contrast, a cross-sectional study in the Netherlands (n = 298) found that the duration of metformin use had no effect (OR, 0.98; 95%CI, 0.87, 1.11).^24^ Similarly, another cross-sectional study found no relationship between metformin use and concentration of cobalamin (β = –0.14; 95%CI, –0.44, 0.16) or holotranscobalamin (β = 0.003; 95%CI, –0.09, 0.09), a marker for cellular cobalamin deficiency.^25^

Because most studies only examined the impact of the dose and duration of metformin treatment, little is known about their cumulative impact on the circulating concentrations of vitamin B_12_. A recent observational study assessed the additive effect of both metformin dose and metformin duration.^31^ In that study, authors used a metformin usage index (MUI), calculated as the daily metformin dose (in milligrams) multiplied by the duration of metformin use (in years), then divide the result by 1000. Interestingly, after multivariable adjustment in logistic regression analysis, MUI was determined to be the most significant predictor of deficiency of cobalamin. Furthermore, the risk for cobalamin deficiency proportionally increased with MUI. Compared with non-metformin users, the highest odds for vitamin B_12_ deficiency were seen in metformin users with MUI >15 (OR, 6.7; 95%CI, 4.4, 10), followed by those who had an MUI >10 (OR, 5.1; 95%CI, 3.1, 8.5). The OR decreased to 1.37 (95%CI, 0.9, 2.2) in individuals who had MUI <5. Hence, the MUI has been proposed as a valid assessment tool to identify people at high risk for cobalamin deficiency and aid in providing appropriate strategies for interventions.

## DISCUSSION

Metformin therapy negatively affected serum vitamin B_12_ concentrations in patients with T2DM. However, the findings related to the association between metformin dose and duration with serum vitamin B_12_ concentrations are inconsistent. These inconsistent observations can be attributed to several factors. One is that there was no agreement on the definition of cobalamin deficiency, which resulted in variations in the cutoff concentrations, rendering their comparisons difficult. Because of variability in immunoassays and measurement methods, serum cobalamin concentration may not be a reliable biomarker of overall cobalamin deficiency. Some studies lacked comparison groups and others were limited by the absence of sufficient data to compare between metformin users and metformin non-users. Furthermore, other sources of heterogeneity across the studies included wide differences in population characteristics and sample size.

### Metformin and vitamin B_12_ status: plausible mechanisms

Metformin affects circulating vitamin B_12_ concentrations through several proposed mechanisms, although these are not fully elucidated.^39–42^ The most plausible mechanism is impaired calcium-mediated uptake of the IF–cobalamin complex to the ileum via the cubilin receptor. Cobalamin absorption occurs in the distal part of the small intestine and is calcium dependent. Here, metformin affects the membrane receptor function by modifying its membrane potential and limiting calcium-dependent vitamin B_12_ absorption. Metformin’s hydrophobic tail attaches to the cell membrane’s hydrophobic core, resulting in a net positive charge that repels calcium cations.^43^ In 1 study, calcium supplementation was shown to reverse vitamin B_12_ malabsorption.^42^

**Figure 1:**
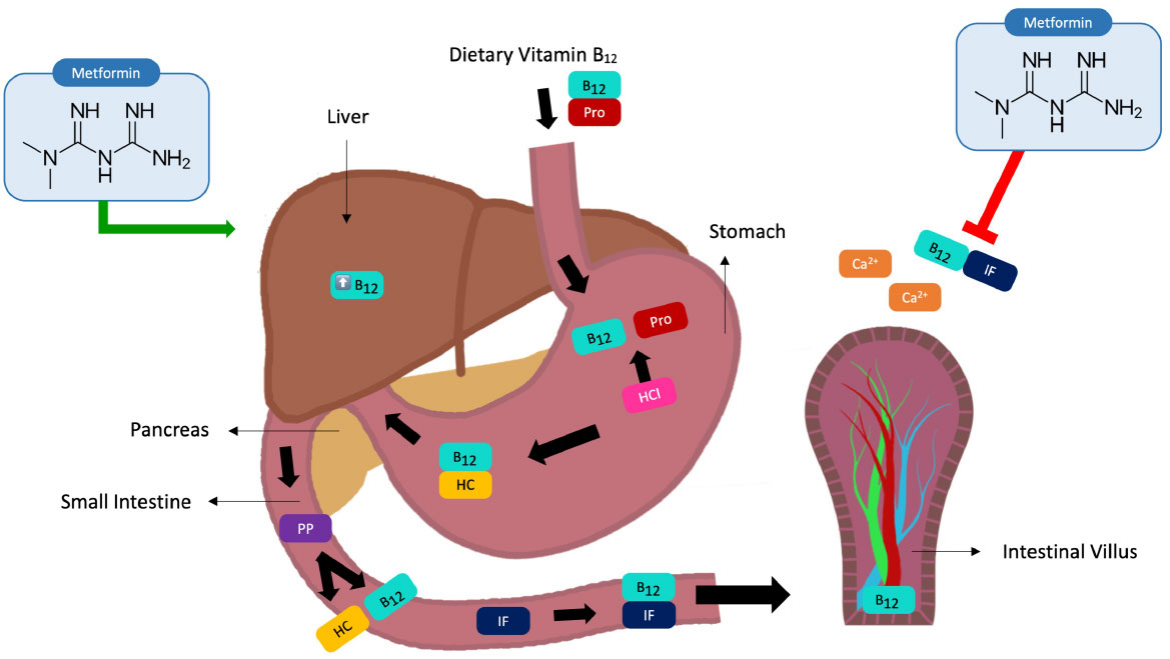
**Metabolism of vitamin B_12_ and proposed mechanisms of metformin-induced vitamin B_12_ deficiency.** The green arrow depicts the vitamin B_12_ accumulation effect of metformin in the liver. The blunted red arrow represents metformin-associated inhibition of calcium-dependent vitamin B_12_ uptake into the ileum. *Abbreviations*: B_12_, vitamin B_12_; Ca^2+^, calcium ion; HC, haptocorrin; HCl, hydrochloric acid; IF, intrinsic factor; PP, pancreatic proteases; Pro, protein; TCII, transcobalamin II.

Another proposed mechanism involves metformin-induced impaired motility of the small intestine. Metformin improves the glucose profile through a series of mechanisms, 1 of which is increased intestinal transit time.^44^ This may alter gut microbiome composition and lead to small intestinal microbial overgrowth. Intestinal bacteria use vitamin B_12_ for metabolic processes, and the resulting metabolites compete with cobalamin absorption, inhibiting the binding of the IF–cobalamin complex to receptors on the ileal mucosa. Another mechanism is that metformin may lower the secretion of IF by gastric cells and reduce concentrations of cobalamin. Additionally, the distribution and metabolism of cobalamin in the tissues may be altered due to metformin-induced cobalamin accumulation in the liver (Figure 1).

### Recommendations

Vitamin B_12_ deficiency results in megaloblastic/macrocytic anemia, paresthesia, cognitive impairment, and other neurological manifestations. Elevated circulating tHcy concentration is an indicator of cardiac disease and inflammation and is observed in those with vitamin B_12_ deficiency.^8^ Although vitamin B_12_–associated hyperhomocysteinemia does not increase cardiovascular disease risk, an increase in cardiovascular disease mortality has been reported in those with T2DM and low serum vitamin B_12_ concentrations, independent of tHcy concentrations.^45^^,^^46^

In light of the associated health consequences of cobalamin deficiency, it is prudent to periodically screen for vitamin B_12_ deficiency in patients who are receiving metformin therapy for T2DM. This is especially important in high-risk populations such as elderly persons, people who take H2 receptor antagonists or proton pump inhibitors, those who practice veganism, those who have undergone partial or total gastrectomy, and patients with malabsorption syndromes.^15^^,^^47–51^ Therefore, vitamin B_12_ deficiency should be corrected while emphasizing the importance of maintaining a prudent lifestyle to improve glycemic control in patients with T2DM.

Furthermore, when screening for deficiency of cobalamin in people with T2DM, it is important to account for both metformin dose and duration. Based on the findings, MUI appeared to be a valid assessment tool that can be incorporated into vitamin B_12_ screening for patients with T2DM who are receiving metformin treatment.^31^ For example, an MUI >5 could serve as the threshold concentration for vitamin B_12_ screening in these patients.

Studies have demonstrated that poor cobalamin status promotes oxidative stress and insulin resistance, contributing to worsening glycemic control and other T2DM outcomes.^8^^,^^52–55^ Because metformin dose and duration are inversely associated with serum vitamin B_12_ concentrations, lifestyle measures focusing on physical activity and prudent dietary practices with concurrent medication use are warranted for a synergistic effect on glycemic control. Physical activity has been associated with a decrease in glycated hemoglobin and medication dosage.^56–58^ Individuals with T2DM should aim for at least 150 minutes of moderate physical activity weekly, with resistance training 2 or 3 times per week, according to the American College of Sports Medicine and the American Diabetes Association.^59^

Diet-based recommendations for patients with diabetes include the consumption of fiber-rich foods (eg, vegetables, fruits, whole grains, legumes), lean meats, and low-fat dairy products, with a focus on foods of low glycemic index or glycemic load.^60^^,^^61^ Often, prudent dietary patterns such as the Mediterranean diet, plant-based diets, and the Dietary Approaches to Stop Hypertension, or DASH, diet are recommended to aid in weight loss and improve blood glucose regulation and insulin sensitivity.^62–66^ Although plant-based diets are beneficial in the management of blood glucose, they are severely deficient in vitamin B_12_.^67^ Therefore, vegans who are receiving metformin therapy should be tested for vitamin B_12_ status periodically. Vegans may also benefit from consuming alternate sources of vitamin B_12_, such as fortified foods, nutritional yeast, fermented nondairy foods, and seaweed.

### Limitations

The main limitation of the reserach is the lack of a gold standard for the assessment of cobalamin status. As a result, investigators have used various biomarkers of cobalamin to define cobalamin status. The most commonly used marker is serum vitamin B_12_ concentration. Serum vitamin B_12_ includes haptocorrin-bound vitamin B_12_ and transcobalamin II–bound vitamin B_12_. Haptocorrin-bound vitamin B_12_, an inactive form, constitutes the majority of total serum cobalamin, whereas transcobalamin II–bound vitamin B_12_, an active form capable of delivering cobalamin to tissues, constitutes a minor portion of total serum cobalamin. Because of this, persons with low normal concentration of serum vitamin B_12_ may have a tissue deficiency. Another marker of vitamin B_12_ status is circulating tHcy. Circulating tHcy concentration lacks specificity because tHcy is elevated not only in vitamin B_12_ deficiency but also in folate, riboflavin, and pyridoxine deficiencies. Another marker of cobalamin status is serum MMA concentration. Serum MMA level is also elevated in kidney dysfunction. Perhaps the best marker of cobalamin status is serum transcobalamin II, because it represents the tissue-deliverable vitamin B_12_. However, it also lacks specificity because, in kidney dysfunction, transcobalamin II level is also elevated. Kidney dysfunction is a common comorbidity associated with diabetes, so in patients with diabetes, the cobalamin status may be overestimated if transcobalamin II, MMA, or tHcy concentrations were used.

Another limitation of the research is the lack of data on how much oral vitamin B_12_ and at what dosage level of metformin should be increased in patients with T2DM. Therefore, based on the current evidence, it is not possible to recommend a very precise amount of dietary vitamin B_12_ for those who are receiving metformin therapy.

## CONCLUSIONS

Evidence for the relationship between metformin use and low serum vitamin B_12_ concentrations is relatively strong. By and large, the data are somewhat consistent, showing an inverse relationship between the dose and duration of metformin use and serum cobalamin concentrations. The deficiency of vitamin B_12_ results in anemia, hyperhomocysteinemia, elevated MMA level, and nerve-related dysfunction, warranting periodic screening for cobalamin deficiency in high-risk groups such as vegans. Healthy lifestyles with medication to improve glycemic control should be emphasized in T2DM management. The potential of lifestyle approaches in lowering metformin dose and improving B_12_ status needs to be further explored.

T2DM mainly afflicts persons of middle age or older. As individuals with T2DM age, the risk of developing suboptimal cobalamin status would also increase because of decreased absorption of cobalamin from the aging gut due to increased gastric atrophy resulting from bacterial overgrowth. Therefore, more studies are needed to establish the dietary vitamin B_12_ allowance for each metformin dose.

There is sufficient evidence in the literature to recommend the use of the MUI as a tool for risk assessment. Also, because of the lack of a gold standard for the measurement of cobalamin nutritional status, clinicians should consider using the MUI as a marker of cobalamin status when screening for cobalamin deficiency in individuals with T2DM.

## References

[1] Watanabe F. Vitamin B12 sources and bioavailability. Exp Biol Med (Maywood). 2007;232:1266–1274. 17959839 10.3181/0703-MR-67

[2] Quadros EV. Advances in the understanding of cobalamin assimilation and metabolism. Br J Haematol. 2010;148:195–204. 19832808 10.1111/j.1365-2141.2009.07937.x PMC2809139

[3] Allen LH , Miller JW, de Groot L, et al Biomarkers of Nutrition for Development (BOND): vitamin B-12 review. J Nutr. 2018;148:1995S–2027S. 30500928 10.1093/jn/nxy201 PMC6297555

[4] Allen LH. Bioavailability of vitamin B12. Int J Vitam Nutr Res. 2010;80:330–335. 21462117 10.1024/0300-9831/a000041

[5] Gröber U , Kisters K, Schmidt J. Neuroenhancement with vitamin B12-underestimated neurological significance. Nutrients. 2013;5:5031–5045. 24352086 10.3390/nu5125031 PMC3875920

[6] Ganji V , Kafai MR. Population reference values for serum methylmalonic acid concentrations and its relationship with age, sex, race-ethnicity, supplement use, kidney function and serum vitamin B12 in the post-folic acid fortification period. Nutrients. 2018;10:74. doi:10.3390/nu10010074. 29329201 PMC5793302

[7] Savage DG , Lindenbaum J, Stabler SP, et al Sensitivity of serum methylmalonic acid and total homocysteine determinations for diagnosing cobalamin and folate deficiencies. Am J Med. 1994;96:239–246. 8154512 10.1016/0002-9343(94)90149-x

[8] Mahalle N , Kulkarni MV, Garg MK, et al Vitamin B12 deficiency and hyperhomocysteinemia as correlates of cardiovascular risk factors in Indian subjects with coronary artery disease. J Cardiol. 2013;61:289–294. 23473764 10.1016/j.jjcc.2012.11.009

[9] Shipton MJ , Thachil J. Vitamin B12 deficiency - a 21st century perspective. Clin Med (Lond). 2015;15:145–150. 25824066 10.7861/clinmedicine.15-2-145 PMC4953733

[10] Oberley MJ , Yang DT. Laboratory testing for cobalamin deficiency in megaloblastic anemia. Am J Hematol. 2013;88:522–526. 23423840 10.1002/ajh.23421

[11] Nexo E , Hoffmann-Lücke E. Holotranscobalamin, a marker of vitamin B-12 status: analytical aspects and clinical utility. Am J Clin Nutr. 2011;94:359S–365S. 21593496 10.3945/ajcn.111.013458 PMC3127504

[12] Hannibal L , Lysne V, Bjørke-Monsen AL, et al Biomarkers and algorithms for the diagnosis of vitamin B12 deficiency. Front Mol Biosci. 2016;3:27. 27446930 10.3389/fmolb.2016.00027 PMC4921487

[13] Yetley EA , Pfeiffer CM, Phinney KW, et al Biomarkers of vitamin B-12 status in NHANES: a roundtable summary. Am J Clin Nutr. 2011;94:313S–321S. 21593512 10.3945/ajcn.111.013243 PMC3127527

[14] O'Leary F , Samman S. Vitamin B12 in health and disease. Nutrients. 2010;2:299–316. 22254022 10.3390/nu2030299 PMC3257642

[15] Langan RC , Goodbred AJ. Vitamin B12 deficiency: recognition and management. Am Fam Physician. 2017;96:384–389. 28925645

[16] Hunt A , Harrington D, Robinson S. Vitamin B12 deficiency. BMJ. 2014;349:g5226. 25189324 10.1136/bmj.g5226

[17] Stabler SP. Clinical practice. Vitamin B12 deficiency. N Engl J Med. 2013;368:149–160. 23301732 10.1056/NEJMcp1113996

[18] Dali-Youcef N , Andrès E. An update on cobalamin deficiency in adults. QJM. 2009;102:17–28. 18990719 10.1093/qjmed/hcn138

[19] Davies MJ , D'Alessio DA, Fradkin J, et al Management of hyperglycemia in type 2 diabetes, 2018. A consensus report by the American Diabetes Association (ADA) and the European Association for the Study of Diabetes (EASD). Diabetes Care. 2018;41:2669–2701. 30291106 10.2337/dci18-0033 PMC6245208

[20] Khan MAB , Hashim MJ, King JK, et al Epidemiology of type 2 diabetes - global burden of disease and forecasted trends. J Epidemiol Glob Health. 2020;10:107–111. 32175717 10.2991/jegh.k.191028.001 PMC7310804

[21] Chapman LE , Darling AL, Brown JE. Association between metformin and vitamin B12 deficiency in patients with type 2 diabetes: a systematic review and meta-analysis. Diabetes Metab. 2016;42:316–327. 27130885 10.1016/j.diabet.2016.03.008

[22] Niafar M , Hai F, Porhomayon J, et al The role of metformin on vitamin B12 deficiency: a meta-analysis review. Intern Emerg Med. 2015;10:93–102. 25502588 10.1007/s11739-014-1157-5

[23] Liu Q , Li S, Quan H, et al Vitamin B12 status in metformin treated patients: systematic review. PLoS One. 2014;9:e100379. 24959880 10.1371/journal.pone.0100379 PMC4069007

[24] de Groot-Kamphuis DM , van Dijk PR, Groenier KH, et al Vitamin B12 deficiency and the lack of its consequences in type 2 diabetes patients using metformin. Neth J Med. 2013;71:386–390. 24038568

[25] Beulens JWJ , Hart HE, Kuijs R, et al Influence of duration and dose of metformin on cobalamin deficiency in type 2 diabetes patients using metformin. Acta Diabetol. 2015;52:47–53. 24908579 10.1007/s00592-014-0597-8

[26] Yousef Khan F , Yousif AB, Suliman A, et al Association of vitamin B12 deficiency with metformin use in patients with type 2 diabetes treated in the largest tertiary care hospital in Qatar. Qatar Med J. 2021;2021:39. 34540601 10.5339/qmj.2021.39 PMC8428509

[27] Miyan Z , Waris N, MIBD Association of vitamin B12 deficiency in people with type 2 diabetes on metformin and without metformin: a multicenter study, Karachi, Pakistan. BMJ Open Diab Res Care. 2020;8:e001151. doi:10.1136/bmjdrc-2019-001151. PMC7252966 32448786

[28] Kim J , Ahn CW, Fang S, et al Association between metformin dose and vitamin B12 deficiency in patients with type 2 diabetes. Medicine (Baltimore). 2019;98: E 17918. 10.1097/MD.0000000000017918 PMC6867725 31725641

[29] Ko SH , Ko SH, Ahn YB, et al Association of vitamin B12 deficiency and metformin use in patients with type 2 diabetes. J Korean Med Sci. 2014;29:965–972. 25045229 10.3346/jkms.2014.29.7.965 PMC4101785

[30] Martin D , Thaker J, Shreve M, et al Assessment of vitamin B12 deficiency and B12 screening trends for patients on metformin: a retrospective cohort case review. BMJ Nutr Prev Health. 2021;4:30–35. 10.1136/bmjnph-2020-000193 PMC8258036 34308109

[31] Shivaprasad C , Gautham K, Ramdas B, et al Metformin Usage Index and assessment of vitamin B12 deficiency among metformin and non-metformin users with type 2 diabetes mellitus. Acta Diabetol. 2020;57:1073–1080. 32266492 10.1007/s00592-020-01526-4

[32] Out M , Kooy A, Lehert P, et al Long-term treatment with metformin in type 2 diabetes and methylmalonic acid: post hoc analysis of a randomized controlled 4.3 year trial. J Diabetes Complications. 2018;32:171–178. 29174300 10.1016/j.jdiacomp.2017.11.001

[33] Aroda VR , Edelstein SL, Goldberg RB et al; Diabetes Prevention Program Research Group. Long-term metformin use and vitamin B12 deficiency in the diabetes prevention program outcomes study. J Clin Endocrinol Metab. 2016;101:1754–1761. 26900641 10.1210/jc.2015-3754 PMC4880159

[34] Infante M , Leoni M, Caprio M, et al Long-term metformin therapy and vitamin B12 deficiency: an association to bear in mind. World J Diabetes. 2021;12:916–931. 34326945 10.4239/wjd.v12.i7.916 PMC8311483

[35] Berchtold P , Bolli P, Arbenz U, et al [Disturbance of intestinal absorption following metformin therapy (observations on the mode of action of biguanides)]. Diabetologia 1969;5:405–412. 5372893 10.1007/BF00427979

[36] Tomkin GH , Hadden DR, Weaver JA, et al Vitamin-B12 status of patients on long-term metformin therapy. Br Med J. 1971;2:685–687. 5556053 10.1136/bmj.2.5763.685 PMC1796258

[37] Rodríguez-Gutiérrez R , Montes-Villarreal J, Rodríguez-Velver KV, et al Metformin use and vitamin B12 deficiency: untangling the association. Am J Med Sci. 2017;354:165–171. 28864375 10.1016/j.amjms.2017.04.010

[38] de Jager J , Kooy A, Lehert P, et al Long term treatment with metformin in patients with type 2 diabetes and risk of vitamin B-12 deficiency: randomized placebo controlled trial. BMJ. 2010;340:c2181. 20488910 10.1136/bmj.c2181 PMC2874129

[39] Andrès E , Noel E, Goichot B. Metformin-associated vitamin B12 deficiency. Arch Intern Med. 2002;162:2251–2252. 10.1001/archinte.162.19.2251-a 12390080

[40] Gilligan MA. Metformin and vitamin B12 deficiency. Arch Intern Med. 2002;162:484–485. 11863489 10.1001/archinte.162.4.484

[41] Andrès E , Goichot B, Schlienger JL. Food cobalamin malabsorption: a usual cause of vitamin B12 deficiency. Arch Intern Med. 2000;160:2061–2062. 10888981 10.1001/archinte.160.13.2061

[42] Bauman WA , Shaw S, Jayatilleke E, et al Increased intake of calcium reverses vitamin B12 malabsorption induced by metformin. Diabetes Care. 2000;23:1227–1231. 10977010 10.2337/diacare.23.9.1227

[43] Raizada N , Jyotsna VP, Sreenivas V, et al Serum vitamin B12 levels in type 2 diabetes patients on metformin compared to those never on metformin: a cross-sectional study. Indian J Endocrinol Metab. 2017;21:424–428. 28553599 10.4103/ijem.IJEM_529_16 PMC5434727

[44] Horakova O , Kroupova P, Bardova K, et al Metformin acutely lowers blood glucose levels by inhibition of intestinal glucose transport. Sci Rep. 2019;9:6156. 30992489 10.1038/s41598-019-42531-0 PMC6468119

[45] van Oijen MGH , Vlemmix F, Laheij RJF, et al Hyperhomocysteinaemia and vitamin B12 deficiency: the long-term effects in cardiovascular disease. Cardiology. 2007;107:57–62. 16763373 10.1159/000093746

[46] Liu Y , Geng T, Wan Z, et al Associations of serum folate and vitamin B12 levels with cardiovascular disease mortality among patients with type 2 diabetes. JAMA Netw Open. 2022;5: E 2146124. 10.1001/jamanetworkopen.2021.46124 PMC8804919 35099545

[47] Loikas S , Koskinen P, Irjala K, et al Vitamin B12 deficiency in the aged: a population-based study. Age Ageing. 2007;36:177–183. 17189285 10.1093/ageing/afl150

[48] Quay TAW , Schroder TH, Jeruszka-Bielak M, et al High prevalence of suboptimal vitamin B12 status in young adult women of South Asian and European ethnicity. Appl Physiol Nutr Metab. 2015;40:1279–1286. 26579949 10.1139/apnm-2015-0200

[49] Mearns GJ , Koziol-McLain J, Obolonkin V, et al Preventing vitamin B12 deficiency in South Asian women of childbearing age: a randomized controlled trial comparing an oral vitamin B12 supplement with B12 dietary advice. Eur J Clin Nutr. 2014;68:870–875. 24736677 10.1038/ejcn.2014.56

[50] Gupta AK , Damji A, Uppaluri A. Vitamin B12 deficiency. Prevalence among South Asians at a Toronto clinic. Can Fam Physician. 2004;50:743–747. 15171677 PMC2214606

[51] Jeruszka-Bielak M , Isman C, Schroder TH, et al South Asian ethnicity is related to the highest risk of vitamin B12 deficiency in pregnant Canadian women. Nutrients. 2017;9:317. 28333089 10.3390/nu9040317 PMC5409656

[52] van de Lagemaat EE , de Groot L, van den Heuvel EGHM. Vitamin B12 in relation to oxidative stress: a systematic review. Nutrients 2019;11:482. 30823595 10.3390/nu11020482 PMC6412369

[53] Giacco F , Brownlee M. Oxidative stress and diabetic complications. Circ Res. 2010;107:1058–1070. 21030723 10.1161/CIRCRESAHA.110.223545 PMC2996922

[54] Meigs JB , Jacques PF, Selhub J, et al; Framingham Offspring Study. Fasting plasma homocysteine levels in the insulin resistance syndrome: the Framingham Offspring Study. Diabetes Care. 2001;24:1403–1410. 11473077 10.2337/diacare.24.8.1403

[55] Setola E , Monti LD, Galluccio E, et al Insulin resistance and endothelial function are improved after folate and vitamin B12 therapy in patients with metabolic syndrome: relationship between homocysteine levels and hyperinsulinemia. Eur J Endocrinol. 2004;151:483–489. 15476449 10.1530/eje.0.1510483

[56] Castaneda C , Layne JE, Munoz-Orians L, et al A randomized controlled trial of resistance exercise training to improve glycemic control in older adults with type 2 diabetes. Diabetes Care. 2002;25:2335–2341. 12453982 10.2337/diacare.25.12.2335

[57] Maiorana A , O'Driscoll G, Goodman C, et al Combined aerobic and resistance exercise improves glycemic control and fitness in type 2 diabetes. Diabetes Res Clin Pract. 2002;56:115–123. 11891019 10.1016/s0168-8227(01)00368-0

[58] Delevatti RS , Bracht CG, Lisboa SDC, et al The role of aerobic training variables progression on glycemic control of patients with type 2 diabetes: a systematic review with meta-analysis. Sports Med Open. 2019;5:22. 31175522 10.1186/s40798-019-0194-z PMC6555839

[59] Kanaley JA , Colberg SR, Corcoran MH, et al Exercise/physical activity in individuals with type 2 diabetes: a consensus statement from the American College of Sports Medicine. Med Sci Sports Exerc. 2022;54:353–368. 35029593 10.1249/MSS.0000000000002800 PMC8802999

[60] American Diabetes Association. 4. Lifestyle management. Diabetes Care. 2017;40(suppl 1):S33–S43. 27979891 10.2337/dc17-S007

[61] Fox CS , Golden SH, Anderson C, et al; American Diabetes Association. Update on prevention of cardiovascular disease in adults with type 2 diabetes mellitus in light of recent evidence: a scientific statement from the American Heart Association and the American Diabetes Association. Diabetes Care. 2015;38:1777–1803. 26246459 10.2337/dci15-0012 PMC4876675

[62] Corsino L , Sotres-Alvarez D, Butera NM, et al Association of the DASH dietary pattern with insulin resistance and diabetes in US Hispanic/Latino adults: results from the Hispanic Community Health Study/Study of Latinos (HCHS/SOL). BMJ Open Diabetes Res Care. 2017;5:e000402. 10.1136/bmjdrc-2017-000402 PMC5530245 28761660

[63] Ahmad S , Demler OV, Sun Q, et al Association of the Mediterranean diet with onset of diabetes in the Women’s Health Study. JAMA Netw Open. 2020;3:e2025466. 33211107 10.1001/jamanetworkopen.2020.25466 PMC7677766

[64] Esposito K , Maiorino MI, Ceriello A, et al Prevention and control of type 2 diabetes by Mediterranean diet: a systematic review. Diabetes Res Clin Pract. 2010;89:97–102. 20546959 10.1016/j.diabres.2010.04.019

[65] Papamichou D , Panagiotakos DB, Itsiopoulos C. Dietary patterns and management of type 2 diabetes: a systematic review of randomized clinical trials. Nutr Metab Cardiovasc Dis. 2019;29:531–543. 30952576 10.1016/j.numecd.2019.02.004

[66] Rock CL , Flatt SW, Pakiz B, et al Weight loss, glycemic control, and cardiovascular disease risk factors in response to differential diet composition in a weight loss program in type 2 diabetes: a randomized controlled trial. Diabetes Care. 2014;37:1573–1580. 24760261 10.2337/dc13-2900 PMC4392939

[67] Dos Santos H , Han D, Perez M, et al Ketogenic vs plantogenic diets for health: A review article. NFS. 2023;53:35–49. doi:10.1108/nfs-11-2021-0344.

